# Insulinoma in pregnancy (a case presentation and systematic review of the literature)

**DOI:** 10.1177/2036361320986647

**Published:** 2021-02-07

**Authors:** Eva M Dobrindt, Martina Mogl, Peter E Goretzki, Johann Pratschke, Agata K Dukaczewska

**Affiliations:** Department of Surgery, Campus Charité Mitte|Campus Virchow-Klinikum, Charité—Universitätsmedizin Berlin, Corporate Member of Freie Universität Berlin, Humboldt-Universität zu Berlin, and Berlin Institute of Health, Berlin

**Keywords:** Insulinoma, hypoglycemia, neuroendocrine tumors, MEN I, endogenous hyperinsulinism

## Abstract

Insulinomas are rare, benign and functional tumors that coincidentally may become overt during pregnancy or in the post-partum period. As the general symptoms of a pregnancy might cover the clinical presentation, diagnosing remains challenging. We present one additional case of a post-partum insulinoma, combined with a systematic review of the literature to sum up relevant details in diagnosis and treatment. A systematic request of Pubmed/Medline was conducted using the following terms: “insulinoma AND pregnancy” and “insulinoma” for a second request of ClinicalTrials.gov. All publications concerning pregnant or post-partum women with insulinoma were included. Thirty-six cases could be identified for analysis. Each publication was reviewed for demographic, diagnostic and therapeutic data. The most frequent clinical signs were unconsciousness and neurological symptoms. 64.9% were diagnosed during early pregnancy and 35.1% post-partum. 91.9% underwent surgery with a third resected during pregnancy without severe influence on fetal or maternal outcome. Three patients died of metastatic disease or misdiagnosing, two of them miscarried. Insulinoma in pregnancy is rare but should be considered in case of unclear hyperinsulinemic hypoglycemia. Surgery can be performed during the second trimester or post-partum with promising outcome.

## Introduction

Insulinomas are small, benign and functional neuroendocrine tumors (NET), descending from neuroendocrine islet cells or multipotent stem cells of the pancreas. The insulin production of these tumors is poorly regulated or totally unregulated by blood glucose levels, leading to spontaneous or post-prandial hypoglycemia.^1^ The incidence is about 1–3 per million per year with a 5-year overall survival rate of 97%.^1–3^ 90–95% of cases are sporadic, affecting slightly more women (60%). 5–10% are associated with a genetic syndrome most frequently with Multiple Endocrine Neoplasia Type 1 (MEN I) and in rarer cases with Von Hippel–Lindau disease, neurofibromatosis 1 or tuberous sclerosis.^1–3^ Insulinomas mainly appear solitarily (90%).^1^ Malignant insulinomas occur in less than 10% and are usually larger (>2 cm) without distinct histological features.^1^ They can only be proven by the presence of metastases.^1^ The average age of onset ranges between the third and fifth decade, while patients with genetic predisposition are often younger.^1,2^

As a seldom peculiarity, insulinomas may coincidentally become overt during pregnancy or in the post-partum period.^4^ As nausea, fatigue, weakness, hypotension and mild hypoglycemia are common during pregnancy and in the early post-partum period, the diagnosis is difficult and the real incidence is potentially underestimated.^4,5^ Currently, only 35 cases have been reported in the literature.^5–38^ Throughout the first trimester, fasting blood glucose concentrations are lower due to an increased insulin level and sensitivity as well as an estrogen and progesterone mediated β-cell hyperplasia, raising its secretion.^6^ Severe hypoglycemia in non-diabetic pregnant women is rare and always suspicious for insulinoma.^6^ During advanced gestation age insulin resistance increases due to placental hormones to provide adequate glucose concentration and ensure the fetal nutritional need.^6^ These physiologic changes explain, why most cases are diagnosed during the first trimester instead of late pregnancy.^5,6^ Hypoglycemia often recurs post-partum, when insulin sensitivity rapidly returns to normal.^5,6^

We present one additional case of a patient diagnosed with an insulinoma post-partum, combined with a systematic review of former case reports to sum up relevant details in diagnosis, medical treatment and optimal time of surgery in these special patients.

## Case presentation

**Figure 1.**
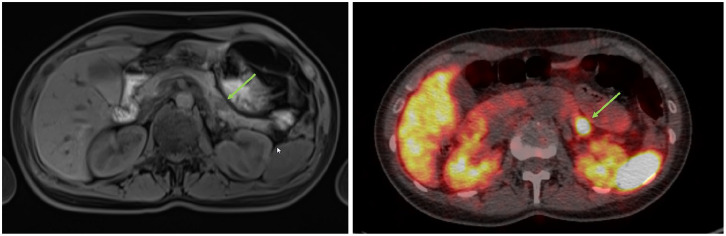
Imaging of our patient, female, 35-years. Left side: MRI scan T1 vibe native, axial. Right side: GA-Dotatoc-PET 3.3 mm, axial. Both imaging methods were performed at an external clinic and provided from the patient who gave her written consent for publishing. The green arrow marks the lesion of the pancreatic tail.

A 35-years old patient, 9 weeks after delivery of her first child presented with reduced consciousness, disorientation and diplopic images. There was no hypoglycemia detected and the patient was examined to rule out epilepsy. Cerebral magnet resonance tomography (MRI) was without a remarkable pathological result. 2–3 days after discharge, the patient developed unconsciousness again with severe hypoglycemia (38 mg/dL). She stabilized after glucose administration and her symptoms were primarily triaged as a dissociative disorder and post-partum depression. After transferal to the department of psychiatry. During sleep deprived she developed another hypoglycemia wherefore a gastroenterologist was consulted. She had a positive fasting test, followed by endoscopic ultrasound (EUS), MRI, and a Positron emission tomography–computed tomography (PET-CT). The EUS detected a hyperperfused lesion 1.13 cm in the pancreatic tail. This could be correlated to MRI and Ga-DOTATOC-PET (Figure 1).

The patient was transferred to our clinic and underwent laparoscopic enucleation of insulinoma. The histopathological report confirmed an 18 mm measuring neuroendocrine tumor with negative surgical margins and no proof of lymphatic or vascular sys, pT1pNxR0L0V0 G1 with a Ki67-index of 2% (negative surgical margins, no lymphatic or vascular system invasion). She immediately continued breastfeeding after surgery without any further hypoglycemic episodes and was discharged after 8 days.

## Materials and methods

**Figure 2.**
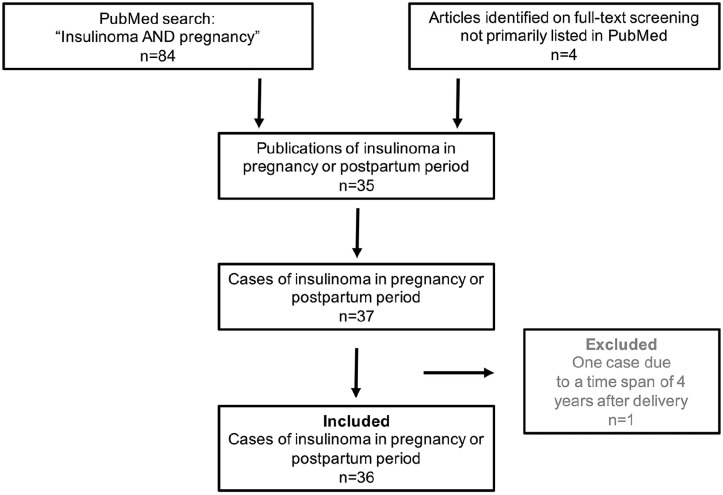
Display shows results of the systematic review of the literature focusing published case reports or case series concerning pregnant or post-partum women diagnosed with an insulinoma.

A systematic Pubmed/medline request was conducted on 1st of April 2020 using the following terms: “insulinoma AND pregnancy.” The second request of ClinicalTrials.gov was performed on 1.04.2020 using the term: “insulinoma.” Including criteria were all presentations of a case report or case series concerning pregnant or post-partum women. Eighty-four items were identified and 31 out of those 84 could be included for further analysis. Four case reports not listed in Pubmed/Medline were found in the literature by an extended data base research. Case 1 from series of Diaz et al was excluded as the time of diagnosis (4 years post-partum) is uncertainly associated with pregnancy.^9^ ClinicalTrials.gov provided 22 items. Five out of those 22 were eligible for insulinoma but none of the trials included pregnant or breastfeeding women. We identified a total of 35 publications with 36 case reports and added our present case for further summary. Schematic display is given in Figure 2.^5–39^ The patient of our case presentation gave written consent for publication. An approval of our Institutional Review Board or Ethics Committee was not needed to conduct this analysis.

Each case was reviewed for demographic data: characteristics of insulinoma, age at diagnosis, time of diagnosis, gestation age, time after delivery, clinical presentation, laboratory findings, positive fasting test, imaging and localization, treatment, surgical technique, time of surgery, glycemic status after surgery, surgical complications, maternal and fetal outcome. Data is given as frequencies in absolute numbers and percentage.

## Results

We identified 36 published case reports and included our patient to further analyze. Demographic and diagnostic data are given in Table 1. Three patients (8.1%) presented with multifocal insulinoma, 1 (2.7%) with additional glucagonoma and 2 patients (5.4%) with liver metastases and a malignant insulinoma. None of the patients were positive for MEN I. Only one showed elevated calcium levels but without reliable proof of MEN I reported. Twenty-four patients (64.9%), were diagnosed during early pregnancy whereas 13 patients (35.1%) became clinically apparent after delivery. Among those diagnosed during pregnancy, the median gestation age was 12 weeks (6-38). Post-partum diagnosis became apparent at a median time of 2 weeks (1–9) after delivery. Most patients presented with unconsciousness (75.7%) and neurological symptoms (64.9%), others with vomiting or nausea (10.8%), psychiatric symptoms (16.2%), sweating (10.8%) or cardiac arrhythmia (5.4%). Hypoglycemia was the most relevant finding and the fasting test positive in 18.9% found. Imaging partly showed high rates of false-negative results for transabdominal ultrasound (100%) and CT (64.7%) and was not consistently conducted.

**Table 1.** Schematic display of clinical characteristics and findings of patients with an insulinoma related to pregnancy.

Demographic data and diagnostics	
Characteristics of insulinomas	37 (100.0%)
Multifocal	3 (8.1%)
Combined with glucagonoma	1 (2.7%)
Malignant insulinoma	2 (5.4%)
Reported MEN I	0 (0.0%)
Mean/median (range) Age at diagnosis (years)	30 (19–47)
Time of diagnosis	
During gravidity; *median Gestation age (weeks)*	24 (64.9%); *12 (6–38)*
Post-partum; *median time after delivery (weeks)*	12 (35.1%); *2 (1–9)*
Clinical presentation	
Nausea, vomiting	4 (10.8%)
Sweating	4 (10.8%)
Disorientation, unconsciousness, syncope	28 (75.7%)
Palpitations, tachycardia, tachyarrhythmia	2 (5.4%)
Neurological symptoms, seizure, grand mal	24 (64.9%)
Psychiatric symptoms	6 (16.2%)
Laboratory findings	
Serum glucose (mg/dl)	27 (3–48)
Insulin (mU/l)	16 (0–687) 43.2
C-peptide (ng/ml)	2.7 (0–10)
Proinsulin (mU/l)	7.0 (0–28)
Positive fasting test	7 (18.9%)
Imaging and localization; *(false negative result)*	
Transabdominal ultrasound	12 (32.4%); *(12 (100.0%))*
Endoscopic ultrasound	11 (29.7%); *(2 (18.1%))*
Magnet resonance tomography	12 (32.4%); *(3 (25.0%))*
Computer tomography	17 (46.0%); *(11 (64.7%))*
GA-DOTATOC-PET	2 (5.4%); *(1 (50%))*
Selective intra-arterial calcium stimulation	9 (24.3%); *(5 (55.6%))*

Data is given in absolute numbers and percentage summarized from the included and presented case reports (n = 37).^5–39^

Data on treatment and outcome are given in Tables 2 and 3. 91.9% underwent surgery with an enucleation as the most frequent procedure. Open surgery was performed in nearly 70% and most of the patients were treated after delivery (67.7%). Normoglycemia was achieved in 69.4%, leaving two patients with the necessary of reoperation. None of the surgically treated patients died. Two developed a pancreatic fistula, one a pancreatitis and one a pancreatic cyst. Those complications could be treated conservatively, and the women did not retain any physical residua. One patient retained severe neurological defects.

**Table 2.** Schematic display of the treatment and complications of patients with insulinoma related to pregnancy.

Treatment and outcome	
Treatment	
Surgery	34 (91.9%)
Enucleation	25 (67.6%)
Left pancreatectomy	6 (16.2%)
Pancreatoduodenectomy	1 (2.7%)
Others	2 (5.4%)
Surgical technique	
Laparoscopy	7 (18.9%)
Laparotomy	25 (67.6%)
No data	2 (5.4%)
Time of surgery	
During gravidity (1.-2. Trimester)	11 (32.4%)
Post-partum	23 (67.7%)
Glycemic status after surgery	
Normoglycemia	26 (76.5%)
Persisting hypoglycemia	2 (5.9%)
Gestation diabetes	1 (2.9%)
Surgical complications	
Pancreatic fistula	2 (5.9 %)
Pancreatitis	1 (2.9%)
Pancreatic cyst	1 (2.9%)
Surgical site infection	1 (2.9%)
Reoperation due to persisting hypoglycemia	2 (5.9%)
Maternal outcome	
Severe neurological defects	1 (2.7%)
Maternal death	3 (8.1%)
Fetal outcome	
Healthy newborn after surgery during pregnancy (*n* = 11)	11 (100.0%)
Breastfeeding (missing data: *n* = 28)	6 (16.2%)
Miscarriage	2 (5.4%)

Data is given in absolute numbers and percentage summarized from the included and presented case reports (n = 37).^5–39^

**Table 3.** Schematic display differential diagnosis of hypoglycemia in pregnant women or during the post-partum period.

Differential diagnosis of hypoglycemia in pregnancy	
During pregnancy	Post-partum
Diabetes mellitus	Diabetes mellitus
Insulinoma	Insulinoma
Proinsulinoma	Proinsulinoma
Non-insulinoma pancreatogenous hypoglycemia syndrome	Non-insulinoma pancreatogenous hypoglycemia syndrome
Post bariatric hypoglycemia	Post bariatric hypoglycamie
Factitious hypoglycemia	Factitious hypoglycemia
Anti-insulin antibodies hypoglycemia	Anti-insulin antibodies hypoglycemia
Medication related hypoglycemia	Medication related hypoglycemia
Paraneoplastic syndrome (e.g. sarcoma)	Paraneoplastic syndrome (e.g. sarcoma)
Adrenal insufficiency	Adrenal insufficiency
Sepsis	Sepsis
Hepatic failure	Hepatic failure
Fructose intolerance	Fructose intolerance
	Sheehan syndrome

Three patients received conservative treatment because of either misdiagnosing an epilepsy (*n* = 1) or severe physical condition because of metastatic disease (*n* = 2). All of those three patients died. The diagnosis in the case with epilepsy was proven by autopsy. Miscarriage was documented in two metastatic cases (5.4%) at 22nd week of gestation several days before the maternal deaths. All patients undergoing surgery during pregnancy gave birth to healthy and well-developed newborns. Successful breastfeeding was reported in six (16.2%) cases with missing data in 28 cases.

## Discussion

### Clinical presentation

Symptoms may be unspecific and appear up to 5 years before diagnosis.^1^ The classic presentation is defined as the “Whipple’s triad”: hypoglycemia, plasma glucose levels < 3 mmol/L (54 mg/dl), and recovery after glucose administration.^2,40^ Hypoglycemia becomes symptomatic with plasma glucose levels below 50 mg/dL (2.8 mmol/L).^8,40,41^ Symptoms can be divided into neuroglycopenic and autonomic symptoms, a physiological response of the adrenergic and cholinergic systems to low plasma glucose.^1–3,40–42^ Additional psychiatric symptoms may lead to severe and prolonged misdiagnosing. Hypoglycemia typically occurs after fasting or exercise but also independently of food intake.^1,40^ The clinical severity is not linked to tumor size or malignancy and can be intermittent but becomes more frequent and persistent over the time.^1,18^ In pregnant women, symptoms may be mistaken as common discomfort during gravidity. 10.8% of the reported cases presented with nausea and vomiting and 75.7% with a loss of consciousness ranging from disorientation to syncope. 64.9% reported about neurological features and our presented patient was initially admitted to a psychiatric department to rule out for post-partum depression. Therefore, a thorough work-up of all unclear hypoglycemic episodes remains substantial in pregnant women and in the early post-partum period, also including potential orphan diseases.

### Differential diagnosis and diagnosing tools

The main differential diagnoses of hyperinsulinemic hypoglycemia are non-insulinoma pancreatogenous hypoglycemia syndrome (NIPHS), post bariatric hypoglycemia, factitious hypoglycemia, anti-insulin antibodies hypoglycemia, medication related hypoglycemia, paraneoplastic syndromes, sepsis and adrenal insufficiency (Table 3).^1,2,40–42^ They are defined by differences in insulin, pro-insulin, and c-peptide levels, characteristics of hypoglycemia and its correlation with food intake, and imaging techniques.

Hypoglycemia and the absence of physiological insulin suppression is suspicious for an endogenous, insulin producing source.^43^ The endogenous hyperinsulinism can be diagnosed by low blood glucose levels (< 55 mg/dL (3.0 mmol/l)), high insulin (⩾ 3 μU/ml (⩾18 pmol/L)) and c-peptide levels (⩾ 0.6 ng/mL (0.2 nmol/L)) and proinsulin determination (⩾5.0 pmol/L).^44,45^ There should be an absence of sulfonylurea in the plasma and/or urine.^44^ When Whipple’s triad is detected, the gold standard for diagnosing insulinoma is the 72-h fasting test. It consists of fasting and the determination of glucose, insulin, c-peptide, proinsulin, and beta-hydroxybutyrate every 6 h or 2 h after the detection of finger prick blood sugar level <59 mg/dL.^44^ The insulin/glucose index and chromogranin A is not recommended for diagnosing.^20^ Glucose levels were low in nearly all case reports with elevated insulin, c-peptide and proinsulin (Table 1). A fastening test was performed and positive in 18.9% (Table 1). It is valid in pregnant women as the suppression of insulin secretion by hypoglycemia is not affected.^5,37^ However, the test can be harmful for mother and child and should be performed under strict supervision.^5,37^

### Imaging and localization

Insulinomas have a mean size between 0.2–2 cm and are equally distributed throughout the pancreas.^46^ Extra-pancreatic lesions are extremely rare (<2%) and can be located in the duodenal wall, the bile duct, Meckel’s diverticulum, ovary, and omentum.^47^ The adequate localization is challenging but necessary, as the only curative strategy is surgery.

Transabdominal sonography is non-invasive and detects insulinomas in 9–66%.^2^ The usage of contrast-enhanced ultrasound raises sensitivity and specificity up to 89.2% and 86.5%.^48^ Nevertheless, 30.6% of the cases received a transabdominal ultrasound but without detection of the tumor (Table 1). The pancreas it not always visible due to overlaying intestine and especially in pregnant women ultrasound can be challenging because of the growing uterus. EUS is one of the most accurate diagnostic techniques and provides additional information on lymph nodes, the possibility of a biopsy or fine-needle aspiration and a sensitivity of 94%.^1–3,49^ It is limited by operator-dependent quality, its invasiveness and a reduced visualization of the pancreatic tail.^50^ 27.8% of the reported women received an EUS with a false-negative rate of 20.0% (Table 1). One of those undetected tumors was located within the pancreatic head and one within the tail. CT has a sensitivity of 83–94% and MRI of 85–95% as especially the small size of insulinomas challenges their detectability by conventional imaging.^2,50^ 27.3% of the reported MRIs and 64.7% of the CT scans were false negative (Table 1). However, even if there have been CTs performed in pregnant women, it should be avoided in pregnancy in nowadays and most centers will conduct an MRI.

Insulinomas have a low proliferation rate and low expression of somatostatin receptor (SSTR) subtype 2. Therefore, PETs using SSTR agents are not useful in benign insulinoma.^51^ In contrast, imaging methods using GLP-1 receptor agents like the 68 Ga-NOTA-MAL-cys40-exendin-4 PET/CT can visualize insulinoma with a sensitivity of 97.7%.^52,53^ There were only two patients with a Ga-DOTATOC-PET, one with an accurate localization and one with a questionable signal close to the jejunum (Table 1). Both women were examined post-partum and a PET should normally be avoided during gravidity.

The Doppman’s test is a selective arterial calcium stimulation test, an invasive procedure consisting of a pancreatic angiography and blood sampling for insulin levels with high sensitivity but reduced clinical relevance due to its invasiveness nowadays.^54^ It was mentioned and performed in former case reports with a false-negative rate of 55.5% (Table 1).

The final diagnosis and indication for surgery can be obtained by the presence of positive fasting test and localization of the tumor in the pancreas with EUS in the majority of patients.^49^

### Treatment options

Medical treatment can be applied until the fetus is matured for symptomatic hypoglycemia in pregnancy, when symptoms are easy to be controlled or if the patient refuses surgery.^4^ However, surgery is the only curative treatment for insulinoma with a cure rate of 77–100%.^55^

Laparoscopic procedures have a comparable success rate with minimal mortality and equivalent safety but should carefully be considered in patients with MEN I and NIPHS due to the risk of multiple lesions.^5,55^ In case of insufficient localization, the pancreas should intraoperatively be examined by bi-manual palpation and intraoperative ultrasound.^56^ Hard tumors, infiltrating behavior or pancreatic duct dilatation are suspicious for malignancy and require a more extensive surgical approach than enucleation.^1,5^ Thirty-four out of 37 patients underwent surgical treatment, with most of them (67.6%) by an open procedure. Most tumors were enucleated, six treated by left pancreatectomy and one by pancreatoduodenectomy. One third were operated during pregnancy and two thirds in the post-partum period. If diagnosis is confirmed before the second trimester, surgery can be performed during the second trimester. Otherwise a bridging symptomatic treatment will be advisable until delivery. Post-operative complications were rare. Only 2 out of 34 patients showed persisting hypoglycemia requiring a second exploration.

In patients not eligible for surgery, metastatic disease, occult insulinoma or those awaiting resection, medical treatment can be administered to control symptoms and tumor progress.^1^ Continuous intravenous glucose infusion might be necessary when dietary adjustments are no longer sufficient.^1^ Diazoxide is the primary treatment option and has a success rate of 50–60% in benign insulinoma.^57^ Somatostatin analogues (SSA), octreotide and lanreotide, can be applied as a second line option in benign or as first line management in malignant insulinomas.^58^ Everolimus is an oral inhibitor of the serine-threonine kinase mammalian target of rapamycin and induces hyperglycemia.^59^ In metastatic disease, streptozotocin with or without 5-fluorouracil or doxorubicin as well as temozolomide with or without capecitabine showed good results to control disease.^60^ Peptide receptors radionuclide therapy (PRRT) is approved for gastroenteropancreatic NETs and has been reported to control hypoglycemia in patients with insulinomas even if the tumor proceeds.^1,58^ There was only one woman, who received octreotide during pregnancy and everolimus after delivery because of metastatic disease. The two other patients who did not undergo surgery received symptomatic therapy. Ethanol ablation or loco-ablative techniques serve as alternative treatment options for non-resectable insulinomas or for liver metastases.^1^

### Outcome

Overall survival after resection ranges from 97% at 5 years to 88% at 10 years and is less than 2 years in case of recurrence.^55^ Recurrence mostly appears within 2.5–3 years and the estimated median disease-free survival is 5 years.^1^ Three patients died of metastatic disease or misdiagnosing and two of them aborted. None of the patients after successful surgery died, reported recurrence and all gave birth to healthy newborns. One patient retained severe neurological defects. Breastfeeding was reported in 16.2%. Nevertheless, after successful treatment there will be no contraindications for breastfeeding.

### Conclusion

Insulinomas in pregnant or post-partum women are rare but should be considered as a differential diagnosis in each case of unclear hyperinsulinemic hypoglycemia. As the clinical presentation varies and invasive examination should be avoided in pregnant women, adequate diagnosis remains challenging. Nevertheless, a glucose fasting test can be conducted even in pregnant women under appropriate supervision. Surgery should be performed during the second trimester or post-partum. Insulinomas are generally benign and maternal and fetal outcome is promising after successful surgery. It would be challenging to conduct large and prospective research on this topic because of the rare incidence. However, it is necessary to be aware and share experience to always implicate modern issues of therapy, diagnostics and progress on this disease to treat patients in the best-known way.
